# Exome sequence analysis identifies a homozygous, pathogenic, frameshift variant in the *MAN2B1* gene underlying clinical variant of α-mannosidosis

**DOI:** 10.3389/fgene.2024.1421943

**Published:** 2024-08-30

**Authors:** Jamil Amjad Hashmi, Muhammad Latif, Reham M. Balahmar, Muhammad Zeeshan Ali, Fatima Alfadhli, Muzammil Ahmad Khan, Sulman Basit

**Affiliations:** ^1^ Department of Basic Medical Sciences, College of Medicine, Taibah University, Madinah, Saudi Arabia; ^2^ Center for Genetics and Inherited Diseases, Taibah University, Madinah, Saudi Arabia; ^3^ School of Science and Technology, Nottingham Trent University, Nottingham, United Kingdom; ^4^ Gomal Centre of Biochemistry and Biotechnology, Gomal University, Dera Ismail Khan, Pakistan; ^5^ Department of Genetics, Medina Maternity and Children Hospital, Medina, Saudi Arabia

**Keywords:** metabolic disorder, *MAN2B1*, alpha-mannosidosis, frameshift variant, developmental delay

## Abstract

**Background:**

α-mannosidosis (MAN) is a rare genetic condition that segregates in an autosomal recessive manner. Lack of lysosomal alpha-mannosidase is the underlying cause of the disease. Symptoms of the disease gradually worsen with the age. Newborns are usually asymptomatic, however, some cases are reported with either congenital ankle equinus or hydrocephalus during the first year. Primary symptoms are characterized by immune deficiency, hearing loss, skeletal abnormalities, progressive mental, motor and speech functions’ impairment followed by facial asymmetry.

**Methods:**

We studied two Saudi families (A and B) with bilateral moderate hearing loss (family A) and clubfoot with glaucoma (family B). Clinical diagnosis was not reached based on phenotype of patients. Therefore, hypothesis-free whole exome sequencing (WES) was performed on DNA samples from affected individuals of both the families, followed by Sanger sequencing and segregation analysis to validate the segregation of the identified variant. Furthermore, 3D protein modelling was performed to determine the *in silico* effects of the identified variant on the protein structure and function.

**Results:**

Re-examination of clinical features revealed that the patients in family A have speech delay and hearing impairment along with craniostenosis, whereas the patients from family B have only clubfoot and glaucoma. WES identified a well known pathogenic homozygous frameshift variant (NM_000528.4: c.2402dupG; p.S802fs*129) in *MAN2B1* in both the families. Sanger sequencing confirmed the segregation of the variant with the disease phenotype in both the families. 3D structural modeling of the MAN2B1 protein revealed significant changes in the tertiary structure of the mutant protein, which would affect enzyme function. This report presents a new case where partial and novel α-mannosidosis phenotypes are associated with a *MAN2B1* gene pathogenic variant.

**Conclusion:**

Patients in both the families have manifested peculiar set of clinical symptoms associated with α-mannosidosis. Family A manifested partial clinical symptoms missing several characteristic features like intellectual disability, dysmorphic features, neurological and abdominal manifestations, whereas family B has no reported clinical symptoms related to α-mannosidosis except the novel symptoms including club foot and glaucoma which has never been reported earlier The current findings support the evidence that biallelic variants of *MAN2B1* are associated with new clinical variants of α-mannosidosis.

## 1 Introduction

*Alpha*-mannosidase (EC# 3.2.1.24) is a lysosomal hydrolase that breaks an α-linked mannose sugar from the non-reducing side of the *N*-linked glycoprotein chain. Deficiency of this enzyme leads to an autosomal recessive lysosomal storage disorder known as α-mannosidosis. This deficiency is caused by a genetic defect in the Mannosidase Alpha Class 2B Member 1 (*MAN2B1*)gene (MIM 248500). Riise et al. (1997) primarily reported the genomic structure of MANB. The MANB gene spans ∼21.5 kb of DNA and comprises 24 exons. The translational product of this gene consists of 1011 amino acids (Riise et al., 1997). Based on recently published data, α-mannosidosis exhibits a broad spectrum of clinical features that can be categorized into three classes. Its severe infantile phenotype (Type I) involves rapid intellectual impairment, hypotonia, hepatosplenomegaly (HSM), severe dysostosis multiplex, and often death betweenthree and 8 years of age. Its milder phenotype (Type II) is manifested by normal early development, intellectual disability during childhood, and good survival into adulthood. A severe form (Type 3) is easily identifiable and characterized by pronounced skeletal deformities and rapid progression, ultimately resulting in untimely demise due to primary central nervous system involvement or myopathy (Thomas et al., 1995; Malm and Nilssen, 2008).

Depending on its symptoms severity and disease onset age, α-mannosidosis has been categorized into specific sub-types as mentioned earlier. Despite being a rare genetic disorder, it can occur in any of the ethnic group round the globe. Its disease frequency in general global population has been found around approximately 1 out of 1000,000 live births (Zielonka et al., 2019). A total of 191 cases have been reported globally in the α-mannosidosis mutation database. The highest number of cases has been reported in Germany, followed by the United States (27 and 22, respectively) (α-mannosidosis mutation database). The most common α-mannosidosis-causing variants are c.2248C>T (p.Arg750Trp) in exon 18, c.1830 + 1G>C (p.Val549_Gluc610del) in intron 14, and c.2426T>C (p.Leu809Pro) in exon 20 (α-mannosidosis mutation database).

A study on 130 unrelated patients with α-mannosidosis from 30 countries revealed 96 distinct pathogenic variantss in *MAN2B1*, including 83 novel variants (Riise Stensland et al., 2012). Although R750W accounted for 27.3% of the disease alleles, it was discovered in 50 patients from 16 different countries. Most of these variants were private. Several therapeutic approaches have been suggested for the treatment of α-mannosidosis, including bone marrow transplantation (BMT) (Walkley et al., 1994; Mynarek et al., 2012a), gene therapy, and enzyme replacement therapy (ERT). BMT has been empirically tested in the context of α-mannosidosis, and the results indicate that it enhances the likelihood of preventing cognitive deterioration and improving associated symptoms. BMT in the cat model has shown promising outcomes and is also a feasible therapeutic option in humans (Mynarek et al., 2012b) however, ERT has shown promising results in smaller animal models, particularly knockout mice (Blanz et al., 2008). The United States Food and Drug Administration (FDA) has recently granted approval of ERT for the use of Lamzede^®^ (velmanase alfa-tycv) as a treatment option for patients with non-central nervous system manifestations of α-mannosidosis, both in adults and pediatric patients (Borgwardt et al., 2013). ERT has demonstrated promising therapeutic potential for α-mannosidosis in the clinical setting (Borgwardt et al., 2018; Guffon et al., 2023).

Owing to a great deal of variation in symptoms severity and disease progression like skeletal and neuromuscular deterioration, α-mannosidosis enjoys a heterogenetic status (Beck et al., 2013a). This broad phenotypic variability coupled with the little availability of discrete clinical symptoms and signs has made it a challenging task to identify α-mannosidosis. The diagnosis of α-mannosidosis is typically made by assessing acid α-mannosidase activity in leukocytes or other nucleated cells, which can be confirmed through a genetic testing approach. Elevated urinary secretion of mannose-rich oligosaccharides may suggest α-mannosidosis, but this is not sufficient for a definitive diagnosis. However, genetic analysis is an essential component of disease diagnosis to explain the nature of the disease, to identify carriers, and to provide support to the affected individuals. Antenatal diagnosis is possible using both biochemical and genetic methods. Management of this condition should be proactive, focusing on preventing complications and treating any manifestations (Berg et al., 1999; Beck et al., 2013b). Owing to clinical variability, patients are generally diagnosed using enzyme-substrate assays in white blood cells (leukocytes). Among therapeutic interventions, enzyme replacement therapy and bone marrow transplantation are still considered the methods of choice for treating the disease condition (Ceccarini et al., 2018). The early diagnosis of the disease is of prime importance owing to the fact that the enzyme replacement therapy is clinically more effective in children then in adults (Borgwardt et al., 2018). One of such early but not congenital features associated with the disease, is hearing loss that alongwith cognitive or learning defects and presence or absence of dysmorphic features, could lead to a potential diagnosis of these lysosomal storage disorders, especially α-mannosidosis.

In this study, we have investigated two Saudi Arabian families to identify possible molecular defects as an underlying cause of the genetic condition. WES in both the families have revealeda pathogenic homozygous frameshift variant (NM_000528.4: c.2402dupG; p.S802fs*129) in *MAN2B1* which may explain the disease phenotype. Sanger sequencing has confirmed the segregation of the variant with the disease phenotypes in both the families. We suggest that correlating the clinical manifestations with the genetic pathways can improve our understanding of the common characteristics of α-mannosidosis. This may facilitate the development of effective therapeutic approaches as well as provide comprehensive care for individuals affected by α-mannosidosis.

## 2 Materials and methods

### 2.1 Patient recruitment

**FIGURE 1:**
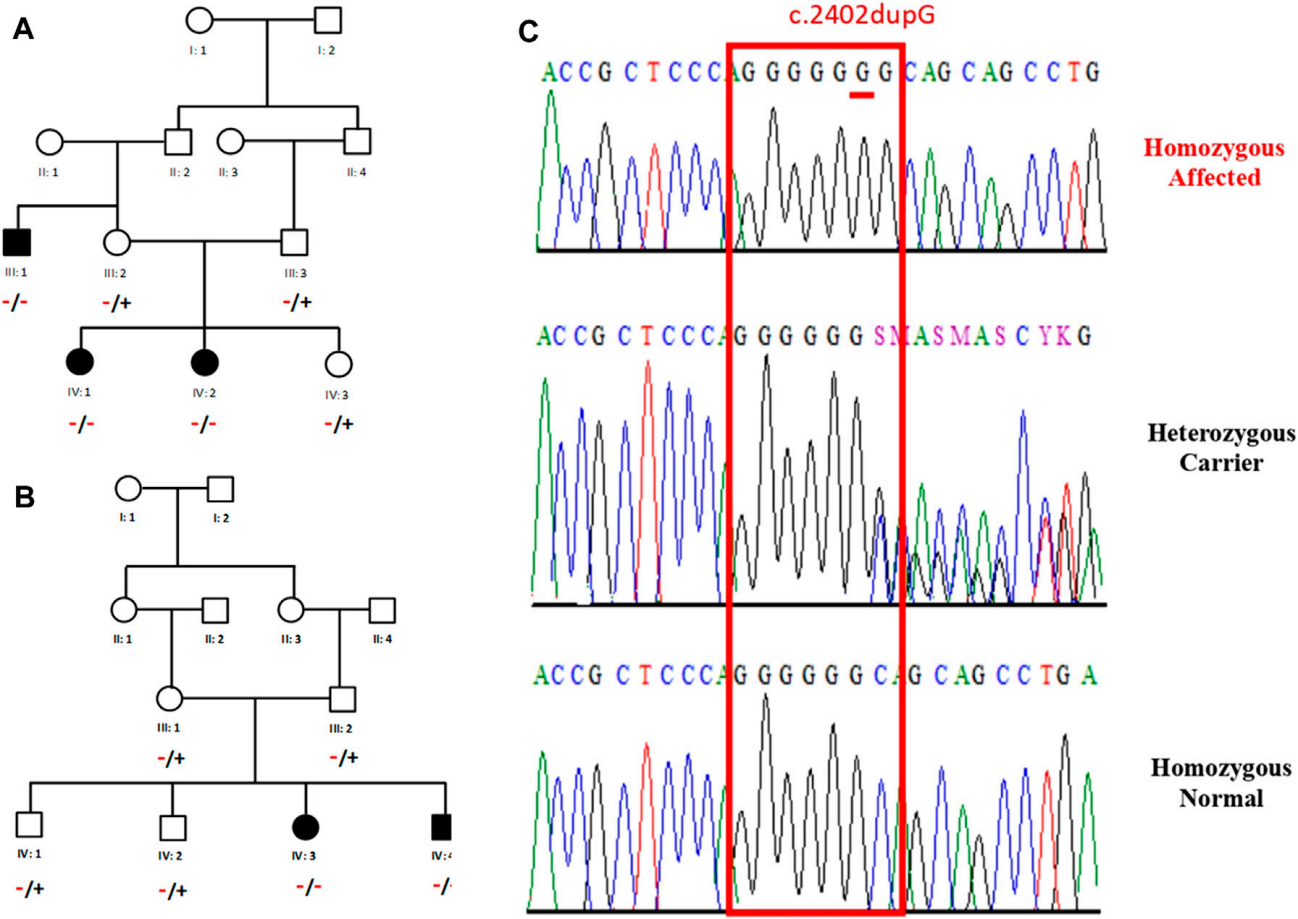
Pedigree of two Saudi families recruited for this study: **(A)**-*Family A*, **(B)**-*Family B*, **(C)**-Sequence chromatograms of affected individuals showing duplication of G at 2402 position.

Two Saudi Arabian families, designated as *Family A* and *Family B*, were recruited at Madinah Maternity and Children Hospital (MMCH) in Madinah, Kingdom of Saudi Arabia (KSA). *Family A* comprised of three affected individuals (III-1, IV-1, and IV-2), while *Family B* had two affected individuals (IV-3 and IV-4). Families originated from the Northern and the Central region of the Madinha province of KSA. This study was conducted in accordance with the guidelines established by the Research Ethics Committee. Approval (TU-21-031, 10-05-2022) was granted by the Ethical Research Committee of Taibah University, Madinah, Kingdom of Saudi Arabia (KSA). The parents provided signed informed written consent after understanding the aims of the study, which were explained to them in the local (Arabic) language. Interviews were conducted with all available family members in both the families, utilizing a questionnaire that facilitated the collection of basic data related to the geographical origin, genealogy, history of miscarriages and newborn deaths, pregnancy course, perinatal suffering, and family history of diseases. Both the families were evaluated clinically and genetically. Primarily, pedigree charts were drawn for both the families using information gathered from their respective parents (Figures 1A,B) followed by collection of blood samples from the available family members. Genomic DNA was extracted from whole blood using a FavorPrep™ Blood Genomic DNA Extraction Mini Kit (Favorgen Biotech Corp., Taiwan) and quantified using a Nano Drop™ spectrophotometer.

### 2.2 Genetic analysis through whole exome sequencing (WES)

Genomic DNA from the affected individuals from each family was subjected to whole exome sequencing (WES). Exome libraries were prepared using the SureSelect Target Enrichment Kit as described previously (Almatrafi et al., 2023; Akbar et al., 2024; Alayoubi et al., 2024; Latif et al., 2024) and paired-end sequencing was performed on an Illumina HiSeq 2500 platform (Illumina, San Diego, CA, United States). After the basic analysis of data file conversion from fastq to BAM, standard filtration steps were followed to analyze the variant calling files (VCF) using the online Illumina BaseSpace analysis tool (https://basespace.illumina.com). Owing to the autosomal recessive pattern of disease inheritance (as evident from the pedigree), priority was given to homozygous and compound heterozygous variants.

### 2.3 Sanger sequencing validation

Sanger sequencing was performed to validate the identified variant in both the families (*A* and *B*). Variant-specific primers were designed using the Primer 3 software (https://primer3.ut.ee/). Bidirectional Sanger sequencing was performed using the BigDye sequencing kit (Ullah et al., 2023; Xiong et al., 2023). Sanger sequencing reads were aligned to the reference sequence using BIOEDIT (https://bioedit.software.informer.com/7.2/). The conservation of amino acids was analyzed using the consurf tool (https://consurf.tau.ac.il/).

### 2.4 Protein modelling and docking analysis

The I-TASSER online tool (Zhou et al., 2022) was employed to construct 3D models for both normal and mutant MAN2B1 proteins, along with their close interactors. The model with the highest confidence score (C-score) was selected for further analysis. Visualization of the designed 3D models was performed using Chimera 1.13.1. Protein-protein docking investigations with close functional interactors were conducted using the Cluspro server (https://cluspro.org).

## 3 Results

### 3.1 Clinical description of patients

*Family A*, that was initially recruited as having hearing imapirment, harbored two female patients aged 9 and 6 years (IV:1 and IV:2) and one male patient aged 15 years (III-1). The patients (IV:1 and IV:2) were born to asymptomatic consanguineous parents, while III:1 was born to non-consanguine parents. Both female patients presented with moderate hearing impairment and speech delay. Upon further investigation from the family, it has been revealed that the birth history of all the patients was unremarkable. Both the female patients (IV:1 and IV:2) congenitally suffered from craniostenosis, whereby patient IV:1 also underwent surgical intervention for this purpose. General clinical examination revealed normal head circumference and body weight relative to those of ethnically matched normal individuals. No HSM or visceral organ defects were observed. Ophthalmic examination revealed a normal fundus, lens, and vision. *Family B* harbored two affected individuals (IV:3 and IV:4) who only suffered from bilateral clubfoot and glaucoma in both eyes with undecended testes (Frank-Ter Haar syndrome -FTHS) in case of IV:4 only. The patients were born to consanguineous parents. Extensive clinical examinations revealed normal physiology of the visceral organs and intellectual function.

### 3.2 Molecular analysis

#### 3.2.1 Exome sequencing and variant interpretation

Exome sequencing was performed using standard methods for a single affected individual from each family. Exome data analysis and variant filtration revealed a homozygous frameshift variant (c.2402dupG; p.S802fs*129) in *MAN2B1* (NM_000528.4; OMIM 248500) in patients from both the families. Bidirectional Sanger sequencing revealed that the identified variant (c.2402dupG) was perfectly segregated with the disease phenotype in both the families (Figure 1C). The variant is reported with a very rare population frequency in multiple public databases including dbSNP, gnomAD, and in-house databases. The variant has been reported as pathogenic in ClinVar (Variation ID: 195465) and dbSNP (rs797044680). Multiple protein sequence alignment has shown that the variant (p.Ser802) is highly conserved across different species and has been predicted to be disease-causing by several bioinformatics tools. Exome data analysis have identified an additional disease causing variant (NM_001308175.2: c.280C>G; p.R94G) in the *SH3PXD2B* gene in the affected individuals of family B. Variants in this gene are responsible for Frank-Ter Haar syndrome (FTHS).

#### 3.2.2 Protein 3D modelling

**FIGURE 2:**
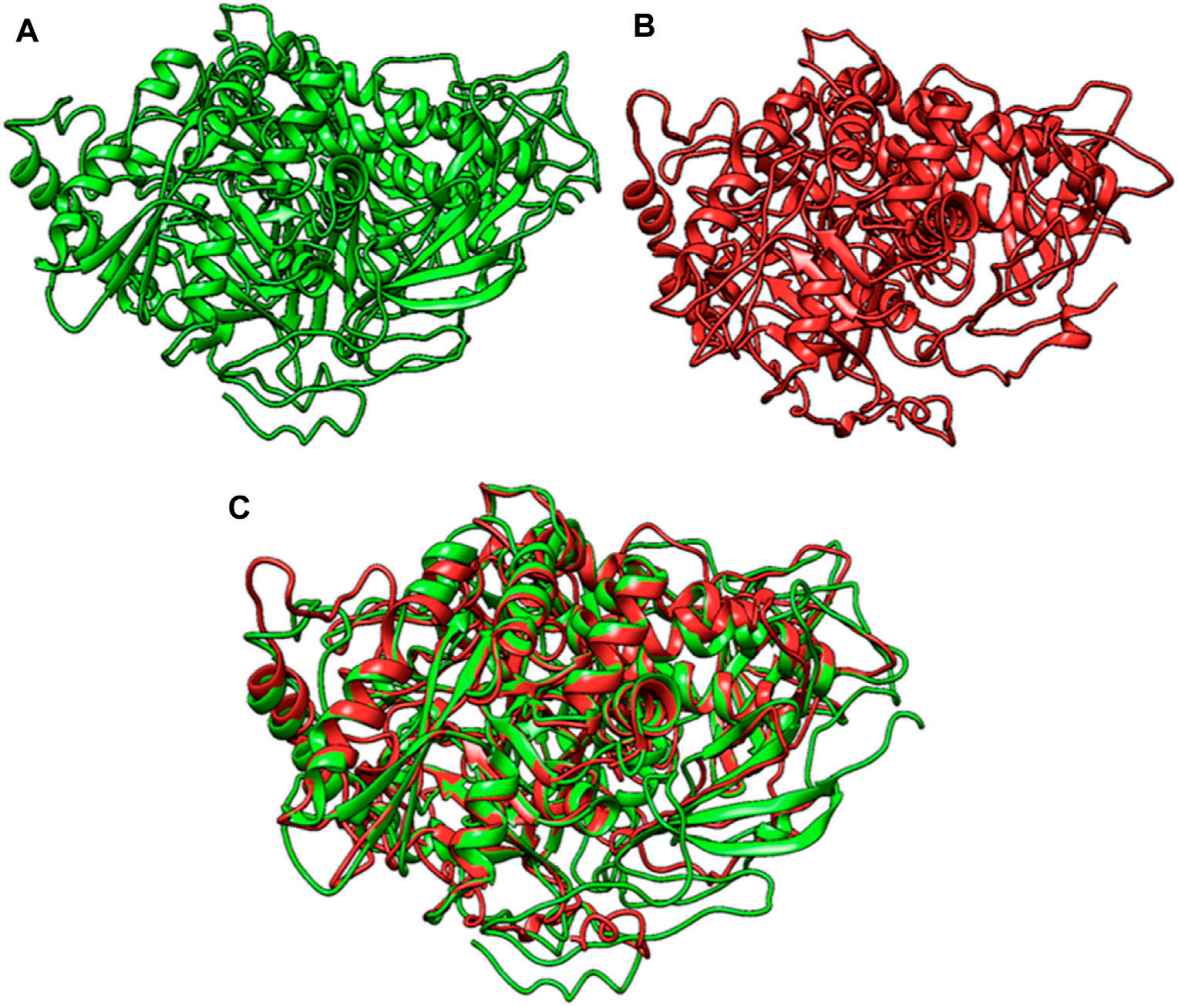
**(A)**-Wildtype MAN2B1 protein, **(B)**-Mutant MAN2B1 protein, and **(C)**-Superimposed structure of wildtype and mutant MAN2B1.

The tertiary structures of the wild-type and mutant MAN2B1 proteins were analyzed and compared, revealing various alterations caused by frameshift variant leading to protein truncation (Figure 2). A comparison of the wild-type and mutant MAN2B1 proteins indicated a similarity index of 78.01% suggesting that the mutant protein is largely different from the wild type protein. Consequently, mutant protein may not perform its enzymatic activity.

#### 3.2.3 Protein docking

**FIGURE 3:**
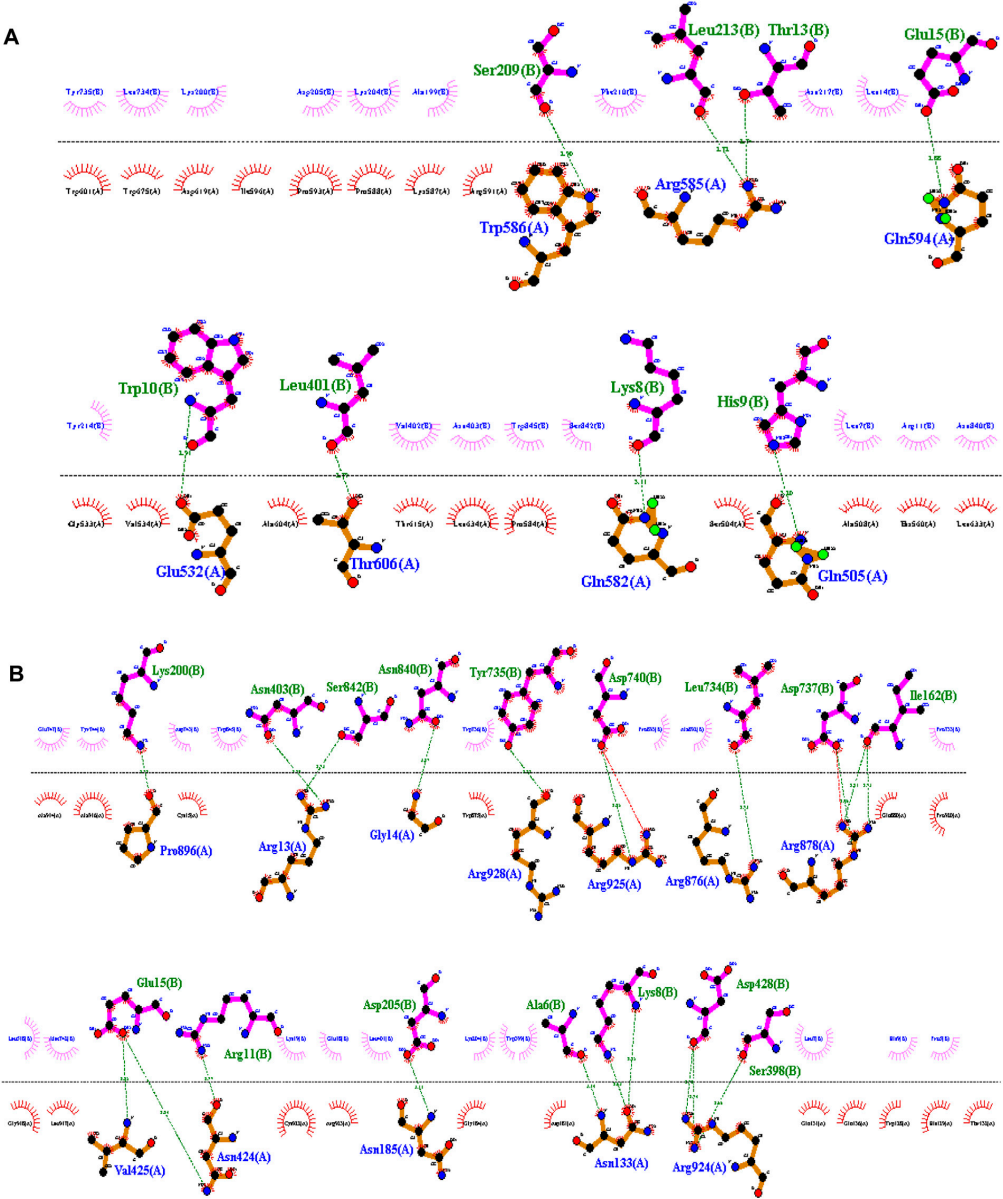
**(A)**-Interactions of wildtype MAN2B1 with MAN2C1, **(B)**-Interactions of mutant MAN2B1 with MAN2C1.

Wild-type MAN2B1 interacts with MAN2C1 via eight hydrogen bonds. The residues of wild-type MAN2B1 involved in this interaction were Trp586, Arg585, Glu594, Glu532, Thr606, Gln582, and Gln505. Mutant MAN2B1 interacts with MAN2C1 via 20 hydrogen bonds and two salt bridges. The amino acid residues of the mutant MAN2B1 protein involved in this interactions were Pro896, Arg13, Gly14, Arg928, Arg925, Arg876, Arg878, Val425, Asn424, Asn185, Asn133, and Arg924 (Figure 3). It is presumed that an increase in interactions may affect various cellular processes, including enzymatic activity, signaling pathways, and protein stability.

## 4 Discussion

Variants in *MAN2B1* causes autosomal recessive α-mannosidosis, a lysosomal storage disease. The clinical spectrum of α-mannosidosis includes intellectual impairment, facial dysmorphism, skeletal anomalies, hearing impairment, neuromotor problems, and immunological disorders. According to a published report, additional clinical manifestations including macroglossia, widely spaced teeth, a large head with a prominent forehead, dysostosis multiplex, flattened nasal bridge, rounded eyebrows, and motor impairment may also appear as a result of *MAN2B1* variants (Malm and Nilssen, 2008). However, no correlation between the different types of variants in *MAN2B1* and the disease phenotype has been established (Borgwardt et al., 2014). Disease-linked mutations are dispersed throughout the gene. Missense mutations are the most prevalent; however, other types of mutations have also been observed, such as nonsense mutations, splice sites, and small deletion mutations. These indel mutations result in an abnormally truncated enzyme or cause the enzyme to assemble incorrectly. These mutations interfere with the ability of the α-mannosidase enzyme to perform its role, causing the accumulation of oligosaccharides in lysosomes and cellular death.

In this report, we present clinical and genetic analysis of two families having multiple affectred members. Family A having 3 affected members (IV:1, IVI:, and III-1) was recruited initially for hearing loss complaint while family B with 2 affected members (IV:3 and IV:4) was initially recruited for bilateral clubfoot and glaucoma as chief symptoms. Family pedigrees showed an autosomal recessive fashion of inheritance (Figures 1A,B). Phenotypes of both families have no common clinical features. Moreover, clinical manifestations were not resembling any known syndrome. Therefore, whole exome sequencing (WES) were performed. WES analysis revealed a homozygous pathogenic variant in *MAN2B1* gene in affected individuals of both families, however, we could not find symptoms of α-mannosidosis in family B. Interestingly, the genetic analysis of family B that was initially recruited for club foot and glaucoma, also revealed that members of family B have a rare genetic disorder Frank-Ter Haar syndrome (FTHS) that is inherited in an autosomal recessive feashion andis hallmarked by the presence of deformities of skeletal, craniofacial and cardiovascular systems. A rare pathogenic homozygous variant c.280C>G; p.R94G) has been identified in the *SH3PXD2B* gene that perfectly segregates with in the family. Careful clinical re-examination of the family revealed that family member IV-4 has undescented testis which is a typical characteristic of FTHS (Khan, 2024). Clinical features of FTHS includes glaucoma as well as bowing of long bones.

The variation in clinical presentation was substantial among the reported cases, particularly with regard to phenotypes (Table 1). Using exome sequencing, we identified a homozygous frameshift variant (c.2402dupG; p.S802fs*129) in *MAN2B1* (NM_000528.4; OMIM 248500) in affected individuals of both the families. The identified variant is a duplication of the G nucleotide that suggestively resulted in a frameshift and subsequently a premature stop codon. According to the ACMG guidelines, the identified variant is ranked as “pathogenic.” The frameshift variant predictably produced a 931 amino acid-truncated protein that has lost its C-terminal domain. The premature termination codon may alter the stability of the mRNA and trigger the degradation of truncated mRNA by the Nonsense Mediated mRNA Decay system. This cannot be further explored due to the unavailability of RNA from the patients. However, this hypothesis is strengthen by the evidence provided by the previous study in which authors revealed a null enzymatic activity in the affected siblings with p.S802fs*129 mutation (Mkaouar et al., 2021). The clinical manifestations of α-mannosidosis have traditionally been categorized into two different systems. The previous system identified a more severe form known as “type I,” which frequently results in death by the age of 8 years and is characterized by infantile onset, rapid mental deterioration, hypotonia, splenomegaly, severe dysostosis multiplex, and severe recurrent infections. According to Ceccarini et al. (2018) and Borgwardt et al. (2014), individuals with the less severe “type II” syndrome exhibit normal early development followed by later childhood development of intellectual disability, hearing loss, coarse facial features, neurologic deterioration, and survival well into adulthood (Borgwardt et al., 2014; Ceccarini et al., 2018). α-mannosidosis is misdiagnosed in Saudi Arabia and throughout the Arab world. Reports have shown that in UAE two distinct variants of the *MAN2B1* gene (c.2119C>T and c.2368C>T) were reported only in two tribes, affecting 5 patients with α-mannosidosis from two families (Al-Jasmi et al., 2013). More recently, Mkaouar et al. (2021) discovered a frameshift duplication p. (Ser802GlnfsTer129) and a missense variant p. (Arg229Trp) in the *MAN2B1* gene in several affected individuals in families (Mkaouar et al., 2021). In Saudi Arabia according to Moammar et al. (2010), a comprehensive screening analysis was conducted on all patients diagnosed with inborn errors of metabolism (IEM) between 1983 and 2008. Surprisingly, the study revealed that two cases from a single family were diagnosed with α-mannosidosis however no genetic analysis was reported. More recently, a case report demonstrated a homozygous variant c.1065delC; p.Ala356fs*7 (NM_001173498.1) in the *MAN2B1* gene (Malibari, 2021). A recent study conducted by Alfadhel et al. (2016). revealed a novel homozygous missense variant, c.1340A > T (p.Asp447Val), in the *MAN2B1* gene, which was found to be consistent with the clinical features of the disease. Additionally, biochemical biomarkers demonstrated a correlation between the genotype and phenotype and showed segregation between the patients and their family members.

**TABLE 1:** Comparison between the genotypic and clinical features of the patients with α-mannosidosis from our study with the previously published ones.

	Description	Mkaouar et al. (2021)	Lehalle et al. (2019)		Our study				
**1**	Genotype	c.2402dupG (p.Ser802Glnfs*129)	c.2402dupG (p. Ser802Glnfs*129) and c.1645-1G>A		c.2402dupG (p.Ser802Glnfs*129)				
		Family 1	Family 1		Family 1 (A)			Family 2 (B)	
**2**	Sex	Male	Female	Male	Patient 1 (Female)	Patient 2 (Female)	Patient 3 (Male)	Patient 1 (Female)	Patient 2 (Male)
**3**	Age (years)	19	10	5	9	6	15	3 years	2months
**4**	Age at diagnosis of AM	18	10	5	-	-	-	-	-
**5**	Mode of Diagnosis	WES	WES	WES	WES	-	-	WES	-
**6**	Hearing impairment age (HI) onset age	ND	8	3	ND	ND	ND	ND	ND
	Level of hearing impairment	ND	Moderate	Moderate	Moderate	Moderate	ND	NO	No
	Speech delay	ND	Absent	Present	Moderate	Moderate	Absent	No	No
**7**	Dysmorphic features	Coarse facial traits	Flat face, ocular proptosis, hypertelorism, and a high palate	Flat face, brachycephaly, hypertelorism, wide forehead, and high palate	Normal	Normal	Normal	Normal	Normal
**8**	Musculoskeletal abnormalities	Thoracic distortions: scoliosis all along the lower vertebrae, pectus excavatum	Skeletal X-Rays normal	Skeletal X-rays normal, Pectus excavatum	Normal	Normal	Normal	Bilateral club foot	Bilateral club foot
**9**	Neurological manifestations	GDD, Microcephaly, Trigonocephaly, Cerebral palsy, Encephalopathy, PVL, Spastic tetraparesis	Cerberal MRI normal, no ataxia, CSS	Cerberal MRI normal, no ataxia				Had surgery for CSS	Mild CSS, no surgery
**10**	Cognitive decline	intellectual disability (Moderate)	No motor delay	No motor delay	Normal	Normal		No	No
**11**	Psychiatric-Behavioral features	ND	Absent	Absent	Absent	Absent	Absent	No	NO
**12**	Immunodeficiency	ND	ND	ND	ND	ND	ND	ND	ND
**13**	Ocular examination	Convergent Strabismus, bilateral AS	Ocular proptosis		Normal	Normal	Normal	Bilateral Glaucoma	BilateralGlaucoma
**14**	Abdominal sonography	Splenomegaly	Heart ultrasound normal, no HM	Heart ultrasound normal, no HM	Normal visceral organs with no HSM	Normal visceral organs with no HSM	Normal visceral organs with no HSM	Normal visceral organs with no HSM	Normal visceral organs with no HSM
**15**	Other features	Haematuria, MBS, cafe’ au lait skin patches	Umbilical hernia, hyperlaxity, Marfanoid habitus	-	-	-	-	Umbilical hernia	Umbilical hernia, undecended testes (FTHS)

^*^ND (Not documented), Bilateral congenital vertical (BCV), Global developmental delay (GDD), Corneal arcus or Arcus senilis (AS), Periventricular leukomalacia (PVL), Craniosynostosis (CSS), hepatomegaly (HM). Hepatosplenomegaly (HSM), Mongolian blue spots (MBS).

We present here, for the first time, an interesting clinical and genetic analyses of two Saudi Arabian families. Due to the extremely variable phenotype of the two families, whole exome sequencing was done and a frameshift pathogenic variant (c.2402dupG) in the *MAN2B1* gene was eventually found in both the families diagnosing α-mannosidosis in both the families. To our surprise, the interesting and the notable point was the manifestation of a new parameter of symptoms associated with the α-mannosidosis with out exhibiting its characteristic reported features. Like, patients in *family A* exhibited moderate hearing impairment, speech delay, and craniostenosis as hallmark features of the disease which is in conformity of other studies also Lehalle et al., 2019. However, patients from family A did not exhibit other characterist symptoms of the disease like intellectual disability, dysmorphic features, splenomegaly and neurological manifestations as reported by Lehalle et al., 2019; Mkaouar et al., 2021. Contrary to this, the patients in *family B* only had glaucoma and clubfoot associated with the α-mannosidosis which has never been reported before. The affected members of *family B* did not show any other charatersitic features related to α-mannosidosis. This variant (c.2402dupG) has been reported in a Tunisian family with severe phenotype (Alayoubi et al., 2024). Intra-familial phenotypic variability due to pathogenic variants in *MAN2B1* gene has been reported by previous studies (Mkaouar et al., 2021). The phenotypic variability due to same pathogenic variant can be attributed to the presence of likely pathogenic variants in additional genes in patients carrying *MAN2B1* variant. Moreover, the genetic background of the patients with *MAN2B1* variants can also affect the expression of phenotype. Furthermore, modifier genes can also affect the manifestation of different phenotypic features in patients with α-mannosidosis.

The sequencing technology has evolved rapidly with the advent of high-throughput next-generation sequencing (NGS). By adopting and leveraging next-generation sequencing tools, clinical laboratories are now performing an ever-increasing catalogue of genetic testing spanning genotyping, single genes, gene panels, exomes, genomes, transcriptomes, and epigenetic assays for genetic disorders. Owing to the increased complexity, this shift in genetic testing has been accompanied by new challenges in sequence interpretation. To the best of our knowledge, this is the first report of a *MAN2B1* variant in tw Saudi families presenting new clinical variants of α-mannosidosis.

## 5 Conclusion

The present study has reported a pathogenic frameshift variant in the *MAN2B1* gene in two Saudi families with α-mannosidosis. The clinical findings of *family A* were consistent to an extent with those of previous studies, for showing hearing impairment, speech delay, craniostenosis but still lacking distinctive disease features like dysmorphic facial features, intellectual disability, hepatosplenomegaly, hypotonia, etc. However, *family B* revealed altogether a totally new phenotype (bilateral glaucoma and clubfoot) that has never been reported earlier, associated with *MAN2B1* gene variant with no other α-mannosidosis related symptoms. The study has also shown that WES has good diagnostic efficiency for mapping diverse genetic disorders. Moreover, genetic testing in the early stages allows for timely diagnosis of the disease, accurate genetic counselling, and eventually, better clinical and therapeutic management. Enzyme-substrate assays should be performed on patient lymphocytes to further explore the disease pathophysiology. Enzyme activity measurements can functionally characterize variants in the *MAN2B1* gene. The extent of the enzyme residual activity due to variants in *MAN2B1* gene can aid in predicting the disease severity and progression.

## Data Availability

The data presented in the study are deposited in the ClinVar repository, accession number SUB14679442.
